# Predicting Microsatellite Instability Status in Colorectal Cancer Based on Triphasic Enhanced Computed Tomography Radiomics Signatures: A Multicenter Study

**DOI:** 10.3389/fonc.2021.687771

**Published:** 2021-06-10

**Authors:** Yuntai Cao, Guojin Zhang, Jing Zhang, Yingjie Yang, Jialiang Ren, Xiaohong Yan, Zhan Wang, Zhiyong Zhao, Xiaoyu Huang, Haihua Bao, Junlin Zhou

**Affiliations:** ^1^ Department of Radiology, Affiliated Hospital of Qinghai University, Xining, China; ^2^ Second Clinical School, Lanzhou University, Lanzhou, China; ^3^ Department of Radiology, Lanzhou University Second Hospital, Lanzhou, China; ^4^ Key Laboratory of Medical Imaging, Lanzhou, China; ^5^ Sichuan Academy of Medical Sciences Sichuan Provincial People's Hospital, Chengdu, China; ^6^ The Fifth Affiliated Hospital of Zunyi Medical University, Zhuhai, China; ^7^ Department of Radiology, Second People’s Hospital of Lanzhou City, Lanzhou, China; ^8^ Department of Pharmaceuticals Diagnosis, GE Healthcare, Beijing, China; ^9^ Department of Critical Medicine, Affiliated Hospital of Qinghai University, Xining, China; ^10^ Department of Biomedical Engineering, Tsinghua University, Beijing, China

**Keywords:** microsatellite instability, colorectal cancer, radiomics, CT, triphasic enhanced phase

## Abstract

**Background:**

This study aimed to develop and validate a computed tomography (CT)-based radiomics model to predict microsatellite instability (MSI) status in colorectal cancer patients and to identify the radiomics signature with the most robust and high performance from one of the three phases of triphasic enhanced CT.

**Methods:**

In total, 502 colorectal cancer patients with preoperative contrast-enhanced CT images and available MSI status (441 in the training cohort and 61 in the external validation cohort) were enrolled from two centers in our retrospective study. Radiomics features of the entire primary tumor were extracted from arterial-, delayed-, and venous-phase CT images. The least absolute shrinkage and selection operator method was used to retain the features closely associated with MSI status. Radiomics, clinical, and combined Clinical Radiomics models were built to predict MSI status. Model performance was evaluated by receiver operating characteristic curve analysis.

**Results:**

Thirty-two radiomics features showed significant correlation with MSI status. Delayed-phase models showed superior predictive performance compared to arterial- or venous-phase models. Additionally, age, location, and carcinoembryonic antigen were considered useful predictors of MSI status. The Clinical Radiomics nomogram that incorporated both clinical risk factors and radiomics parameters showed excellent performance, with an AUC, accuracy, and sensitivity of 0.898, 0.837, and 0.821 in the training cohort and 0.964, 0.918, and 1.000 in the validation cohort, respectively.

**Conclusions:**

The proposed CT-based radiomics signature has excellent performance in predicting MSI status and could potentially guide individualized therapy.

## Introduction

Colorectal cancer (CRC) is characterized by complex biological features and shows distinct heterogeneity. Even though the clinicopathological characteristics of CRC are similar, there is still significant variability in treatment response and prognosis (1). Two major molecular events are involved in the occurrence and development of CRC (2, 3). The vast majority of CRCs are caused by chromosomal instability events (approximately 85%), including mutations in *APC*, *KRAS*, and *TP53* genes, etc. However, a small percentage of CRCs are caused by microsatellite instability (MSI) (approximately 15%).

Mismatch repair (MMR) genes are highly conserved and are involved in repairing DNA base mismatches. They are beneficial in maintaining genome stability and reducing spontaneous mutations (4). MMR proteins include MLH1, MSH2, MSH6, and PMS2. During DNA replication, minor DNA mismatches occasionally occur, which are recognized by these proteins and then cut and synthesized into new strands for repair (5, 6). When any one of these four proteins are non-functional, they cause accumulation of DNA base mismatches in proliferating cells, a phenomenon known as MSI (6).

MSI status is currently a key predictor for evaluating the treatment strategies and prognosis of CRC patients (7, 8). Compared with microsatellite-stable (MSS) CRC patients, CRC patients with MSI status are more likely to benefit from immunotherapy, but they do not benefit from pyrimidine analogs or fluorouracil-based adjuvant chemotherapy (9–11). In addition, CRC patients with MSI status may have a favorable prognosis (12–14). The National Comprehensive Cancer Network (NCCN), European Society for Medical Oncology (ESMO), and Japanese Society for Cancer of the Colon and Rectum (JSCCR) guidelines recommend testing the MSI status of CRC patients (4, 15, 16).

At present, MSI status detection is mainly done through immunohistochemistry (IHC) and polymerase chain reaction (PCR) methods on biopsy or surgical tissue, both of which are invasive and costly (8, 17). Furthermore, the small part of the tissue captured by biopsy may not be sufficient to accurately reflect the MSI status of tumors (18, 19). In addition, these advanced biological tests can only be performed in qualified tertiary medical centers, as local medical institutions have not widely adopted these methods because of the lack of suitable medical equipment (20). Therefore, developing a non-invasive, cost-effective, and easily repeatable method to identify MSI status could help clinicians to develop more accurate treatment strategies for CRC patients.

Radiomics is a burgeoning field in the era of precision medicine, involving screening, diagnosis, treatment, and prognostic assessment of multiple systemic diseases (21–24). By extracting high-dimensional, mineable data from medical imaging and evaluating its association with clinicopathologic factors or gene expression, radiomics facilitates the formulation of individualized treatment strategies. Radiomics has been widely used in CRC stage assessment (21), tumor differentiation identification (25), post-neoadjuvant chemotherapy efficacy evaluation (26), and *KRAS* mutation status identification (27). A previous study demonstrated a significant correlation between a CT-based radiomics signature and MSI status in CRC patients (28, 29). These results indicate that pretreatment CT may be associated with MSI status and that radiomics analysis may greatly contribute to MSI status identification. However, previous studies have only included a single group and lack external validation. Moreover, the superiority of the venous phase (VP) compared to arterial and delayed phases (AP and DP, respectively) in the prediction of MSI status in CRC patients remains to be confirmed. Therefore, the aim of this study was to investigate whether a CT-based radiomics signature could identify MSI status in CRC patients and to identify the phase with the most robust and high-performing radiomics signature from triphasic enhanced CT.

## Materials and Methods

### Patients

**Figure 1:**
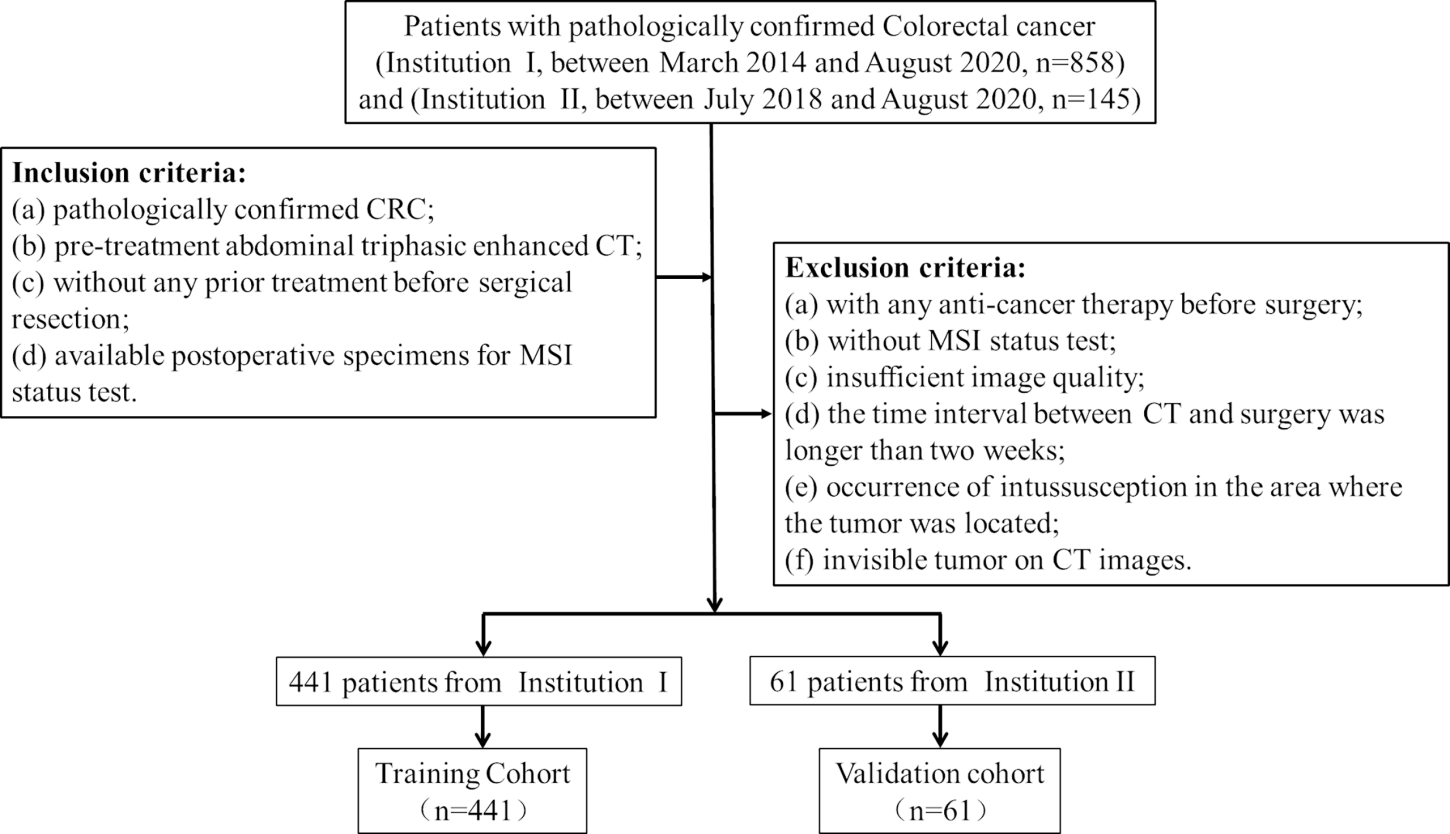
Patient inclusion and exclusion details and the patient recruitment pathway.

Ethical approval was obtained by the medical ethics committee in both participating centers (center I: Lanzhou University Second Hospital; center II: The Second People’s Hospital of Lanzhou city), and the requirement for informed consent was waived due to the retrospective nature of the study. Patient inclusion and exclusion details and the patient recruitment pathway are shown in **Figure 1**. The institutional database in center I was searched for eligible patients who underwent curative resection between March 2014 and August 2020, and a total of 441 consecutive patients were enrolled. This study included 255 males (42.2%) and 186 females (57.8%), with an average age of 58.64 ± 12.92 years (range, 20–89 years). Furthermore, 61 patients from center II were also enrolled between July 2018 and August 2020, including 38 males (62.3%) and 23 females (37.7%), with an average age of 56.93 ± 11.94 years (range, 27–84 years). The model for MSI prediction was established in the training cohort and evaluated in the external validation cohort. The baseline clinical data of all CRC patients, including age, sex, tumor location, carcinoembryonic antigen (CEA) level, CA125 level, and CA199 level, were collected. Two radiologists (radiologist A, Y.T.C.; radiologist B, J.Z.) with more than 10 years of experience in abdominal imaging collected radiological features on preoperative CT images and recorded the results, including clinical tumor/lymph node (cT/N) stage and tumor maximum diameter (maximum diameter perpendicular to the long axis of the cross-sectional image). In order to minimize the deviation of the measurement results, the quantitative data was taken as the final result by the average of the measurement values of the two radiologists, while the qualitative data is diagnosed by the two radiologists independently, and the disagreement is resolved through negotiation.

### Identification of MSI Status

MSI status was evaluated by immunohistochemical staining of MMR proteins (MLH1, MSH2, MSH6, PMS2). The standard streptavidin biotin-peroxidase procedure was performed on postoperative tissues to identify the MSI status. Patients were classified into the MSI or MSS group according to the staining results of MMR proteins. Among the four MMR proteins, negative staining for one or more proteins was defined as MSI. MSS was defined as positive staining for all four MMR proteins (6).

### CT Image Acquisition and Segmentation

All patients underwent abdominal and/or pelvic enhanced CT scans in two institutions, which covered the whole tumor. Triphasic enhanced CT images were retrieved from the picture archiving and communication system (PACS, Carestream; Rochester, NY) and stored in corresponding folders in DICOM format for further analysis. The CT scanner and acquisition parameters of the three institutions are listed in **Supplementary Tables S1, S2**.

Two gastrointestinal radiologists (radiologist A and radiologist B) performed three-dimensional (3D) radiomics segmentation on AP, VP, and DP using ITK-SNAP software (version 3.6.0; www.itksnap.org). Radiologist A segmented 300 cases and radiologist B segmented the other 202 cases.

For radiomics segmentation, an ROI was manually delineated on each slice of the tumor. Air and feces in the intestinal tract and pericolonic fat were carefully excluded from the contours. Finally, three ROIs (AP, DP, and VP) were generated for each patient. To evaluate inter-observer reproducibility and robustness of feature extraction, radiologist A and radiologist B randomly selected 30 patients and performed manual segmentation again. We estimated the reproducibility of feature extraction using inter-class correlation coefficients (ICCs), where ICCs greater than 0.80 indicated good reproducibility (30). Additionally, 30 patients were randomly selected from each CT scanner to build the CT scanner set for calculating intra- and interclass correlation coefficients (ICCs).

### Feature Extraction

Before feature extraction, we adopted three steps to preprocess the CT images. First, we resampled images to 1 mm × 1 mm × 1 mm using linear interpolation to try to reduce the influence of different layer thicknesses. Second, we transformed the continuous images into discrete integer values using gray-level discretization processing (bin width = 25). Finally, Laplacian of Gaussian (LoG) and wavelet image filters were used to eliminate mixed noise in the processing of image digitization and to obtain low- or high-frequency features.

Radiomics features were extracted using the PyRadiomics package (31). Seven classes of radiomics features were extracted from the original and filtered images (wavelet and LoG). Finally, 1037 3D radiomics features were extracted from each patient. The feature types and their numbers are as follows: (1) first-order (histogram) features (n = 198); (2) shape features (n = 14); (3) gray-level co-occurrence matrix (GLCM) features (n = 264); (4) gray-level run-length matrix (GLRLM) features (n = 176); (5) gray-level size zone matrix (GLSZM) features (n = 176); (6) neighboring gray-tone difference matrix (NGTDM) features (n = 55); (7) gray-level dependence matrix (GLDM) features (n = 154). The specific definitions and descriptions of the features are demonstrated in the **Supplementary Materials**.

### Features Selection and Prediction Model Building

After radiomics feature extraction, all missing data in the training cohort were replaced by median value, z-score normalization was performed on each feature, and the same preprocessing procedure was applied to the validation cohort. We performed a binary classification task for MSI status prediction: MSS vs. MSI expression. It is worth noting that the sample numbers of the two groups were unbalanced between the training and validation cohorts. The initial bias adjustment method was used to correct the influence of unbalanced sample size. The adjustment bias *b*_0_ was determined using the following equation:

$$p_{0}=\frac{pos}{\left(pos+neg\right)}=\frac{1}{1+e^{-b_{0}}}$$

$$b_{0}=-\log_{e}\frac{1}{p_{0}-1}$$

The process of radiomics feature selection that is most related to MSI status consists of three steps. First, univariate analysis with the Mann-Whitney U test was performed for feature selection to retain features with p < 0.05 for the subsequent process. Second, the least absolute shrinkage and selection operator (LASSO) method was used to retain features closely associated with MSI status. Finally, multivariable stepwise logistic regression further eliminated irrelevant features and retained the most informative features. A ten times five-fold cross-validation method was applied to avoid overfitting and to identify the model with the best performance.

Three radiomics models were established based on the above radiomics signatures in triphasic phase-enhanced CT images (APR, VPR, and DPR models). In order to verify whether the model combining the triphasic enhanced phases can improve the prediction performance of MSI status, the FR model was built based on AP, VP, and DP fusion features from 3D segmentation patterns. The maximum area under the curve (AUC) in the training cohort determined the final regularization parameter. Furthermore, the Radiomics models predicted a radiomics signature demonstrating the likelihood of MSI status for each patient.

### Clinical, Combined Model, and Nomogram Construction

For clinical and radiological features, the chi-squared test or Fisher’s exact test was used to compare differences in sex, CEA, CA125, CA199, cT stage and cN stage, while the Student’s t-test or Mann-Whitney U test was used to compare differences in age, and maximum diameter between the MSS and MSI groups in the training and external validation cohorts. Generally, P-values < 0.05 (two-sided) were considered statistically significant. We performed multivariable analyses to identify the most important features. A clinical model was established based on the inclusion of selected features.

A combined model (clinical Radiomics) was developed based on correlated clinicalradiological features and radiomics features to verify whether the combination of radiomics signatures and clinicalradiological features could improve the prediction of MSI status, and it was presented as an individualized nomogram.

**Figure 2:**
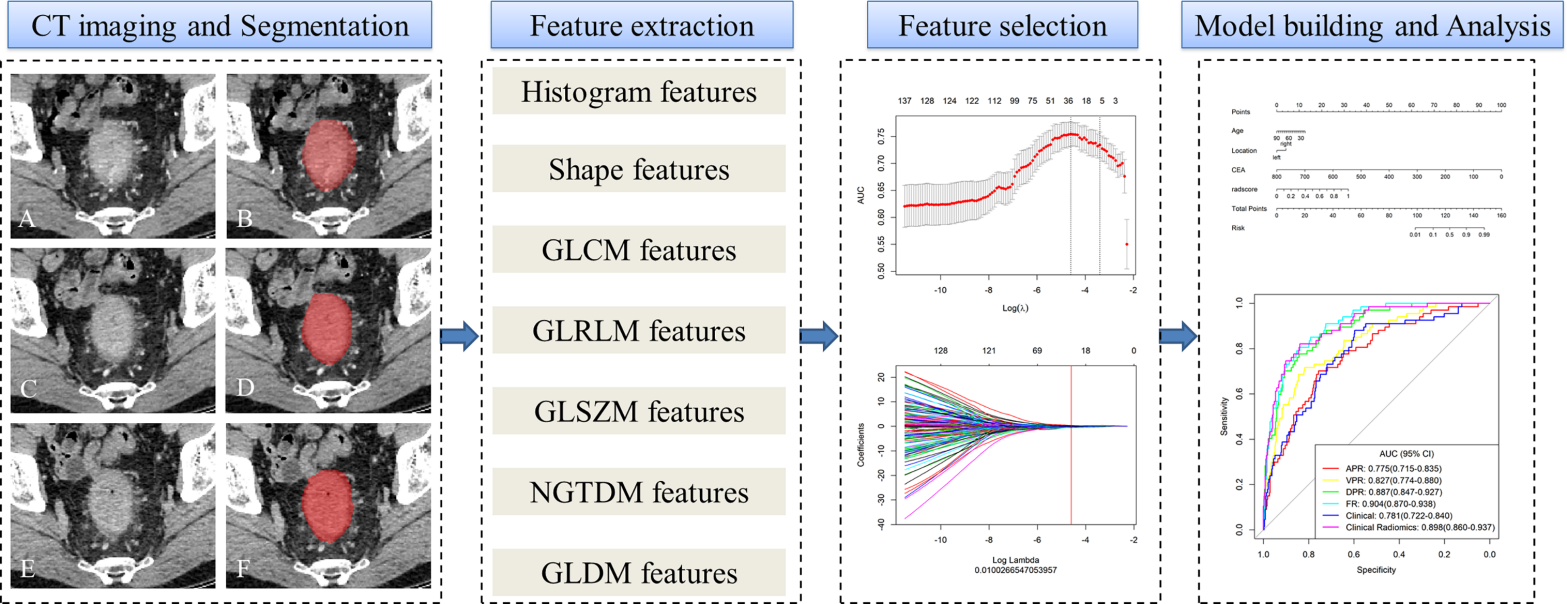
Workflow of microsatellite instability (MSI) prediction building and analysis. The tumors were segmented on arterial phase **(A, B)**, delayed phase **(C, D)** and venous phase **(E, F)** CT images to form volumes of interest (VOIs). One thousand and thirty-seven quantitative radiomics features were extracted from each patient. The least absolute shrinkage and selection operator (LASSO) was used to select the features. Multivariate logistic regression was used to build radiomics, clinical, and clinicoradiomics combined models for MSI prediction. Finally, the radiomics signature and clinical factors were incorporated into a nomogram for individual evaluation. Receiver operating characteristic curves were used to evaluate the clinical usefulness of the nomogram.

Using multivariate logistic regression coefficients, a nomogram incorporating clinicalradiological characteristics and radiomics signatures was created for the training and external validation cohorts following the selection of clinical characteristics and model comparison. This nomogram provides a more convenient and reliable tool for patients and clinicians. A flowchart of the study is shown in **Figure 2**.

### Statistical Analyses

All statistical analyses were conducted using the R statistical software package (version 3.6.3; http://www.Rproject.org). Student’s t-test, the Mann-Whitney U test, and the chi-squared test or Fisher’s exact test were used to compare continuous and categorical variables, as appropriate. A two-sided P-value < 0.05 was considered statistically significant. ICCs were used to calculate the consistency of measurements between the two radiologists and different CT scanners. Receiver operating characteristic (ROC) analysis was used to evaluate the predictive accuracy of the different models. The AUC, 95% confidence interval (CI), accuracy, sensitivity, specificity, positive predictive value (PPV), and negative predictive value (NPV) were calculated for each model. Precision-recall (PR) curves and the DeLong test were used to compare the AUC estimates of the discrimination performance between different predictive models. A calibration curve was constructed to assess the goodness-of-fit of the models. The Hosmer-Lemeshow (HL) test was performed to assess the agreement between the predicted MSI status and the observed outcomes. To verify the clinical usefulness of the models, we quantified the net benefit at different threshold probabilities in the dataset using decision curve analysis (DCA).

## Results

### Clinical Characteristics

Patient and tumor characteristics in the training cohort are listed in **Table 1**. This study included 502 CRC patients (441 patients in center I, 61 patients in center II) in the final analysis. The prevalence of MSI was 15.19% (67/441) in center I and 14.75% (9/61) in center II.

**Table 1:** Characteristics of patients in the training cohort [median (Q1, Q3) or no. (%)].

Characteristics		Training cohort (n=441)		
		MSS	MSI	P value
Age (years)		61.00 (51.00, 68.00)	51.00 (42.50, 63.00)	<0.001
Gender	Female	153 (40.9%)	33 (49.3%)	0.203
	Male	221 (59.1%)	34 (50.7%)	
Tumor Location	Left	267 (71.4%)	22 (32.8%)	<0.001
	Right	107 (28.6%)	45 (67.2%)	
CEA level		4.03 (2.18, 12.82)	2.81 (1.60, 6.37)	0.009
CA125 level		12.02 (8.73, 17.30)	16.71 (9.59, 24.64)	0.004
CA199 level		13.45 (7.74, 26.59)	9.99 (5.94, 25.36)	0.067
cT stage	T1	12 (3.2%)	0 (0.0%)	0.671
	T2	58 (15.5%)	10 (14.9%)	
	T3	236 (63.1%)	47 (70.1%)	
	T4	68 (18.2%)	10 (14.9%)	
cN stage	N0	210 (56.1%)	44 (65.7%)	0.201
	N1	81 (21.7%)	11 (16.4%)	
	N2	83 (22.2%)	12 (17.9%)	
Maximum diameter (mm)		19.80 (15.71, 25.62)	24.70 (18.31, 30.80)	0.001

### Predictive Performance of the Clinical Model

Age, tumor location, CEA level, CA125 level, and maximum diameter were found to be significantly different (P < 0.05) between the MSI and MSS groups in the training cohort, but other characteristics were not significantly different (P > 0.05). Finally, after multivariate analyses, age, tumor location, and CEA were selected as independent predictors of MSI and were enrolled into the clinical model (**Supplementary Table S3**). The clinical model showed moderate performance in predicting MSI both in the training cohort and the validation cohort, with an AUC of 0.781 (95%CI, 0.722-0.840) in the training cohort and 0.919 (95%CI, 0.833-1.000) in the validation cohort (**Table 3**). The accuracy, sensitivity, and specificity were 0.721, 0.716, and 0.722 in the training cohort and 0.869, 0.889, and 0.865 in the validation cohort, respectively.

### Radiomics Signature Building and Discrimination Performance Assessment

**Figure 3:**
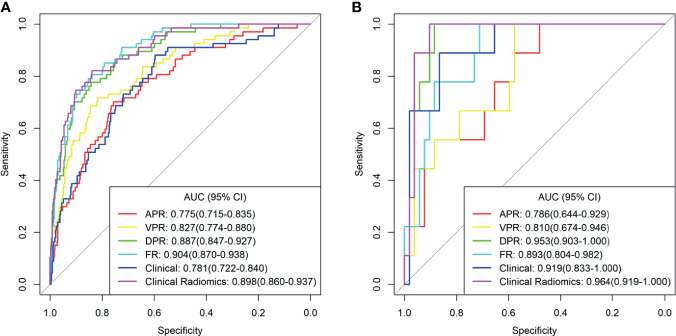
The receiver operating characteristic (ROC) curves of the different models in training cohort **(A)** and validation cohort **(B)**. AUC, area under the curve; APR, radiomics model of arterial phase; DPR, radiomics model of delayed phase; VPR, radiomics model of venous phase; FR, radiomics model of fusion of arterial phase, delayed phase and venous phase features; Clinical Radiomics, fusion of clinical risk factors and radiomics features of delayed phase.

ICCs were calculated to evaluate the agreement of features extracted by the two radiologists and different CT scanners, and ICC values > 0.80 indicated good agreement. A total of 1037 3D radiomics features from AP, VP, and DP images were extracted for each patient, and irrelevant features were removed as described earlier. Finally, 6 AP, 10 VP, and 16 DP 3D radiomics features were retained as the final signatures. The feature names and distributions are listed in **Table 2**. The values of these features were significantly different between the MSI and MSS groups. Following stepwise regression analysis, 16 features were selected after fusion of the radiomics features from AP, VP, and DP (FR model). Significant differences were found in these features between the MSI and MSS groups (**Supplementary Figure 1**). As shown in **Supplementary Figure 2**, the feature heatmaps show that the correlation between most of the features is below than 0.9, indicating that the final features are less collinear with each other. Four models were built based on the above radiomics signatures for preoperatively predicting MSI (APR, DPR, VPR, and FR models). The AUC, accuracy, sensitivity, specificity, PPV, and NPV for each model are listed in **Table 3** and **Figure 3**. The DPR model had optimal predictive performance compared to APR or VPR in the training and validation cohorts (**Figures 3A, B**). In addition, the FR model had a higher predictive AUC than APR, DPR, or VPR in the training cohort. In the validation cohort, the FR model had a higher predictive AUC than APR or VPR in the training cohort but slightly lower than the AUC of the DPR model.

**Table 2:** The final signatures selected from 3D radiomics features.

Arterial phase (n=10)	Venous phase (n=10)	Delayed phase (n=16)	Radiomics (n=16)
A_original_glszm_GrayLevelVariance	V_original_glszm_GrayLevelVariance	D_original_shape_Elongation	V_original_glszm_ZoneEntropy
A_log.sigma.5.0.mm.3D_glszm_LargeAreaHighGrayLevelEmphasis	V_original_glszm_ZoneEntropy	D_original_firstorder_Range	V_wavelet.LHL_glszm_LargeAreaHighGrayLevelEmphasis
A_wavelet.LHL_firstorder_90Percentile	V_log.sigma.5.0.mm.3D_gldm_DependenceNonUniformityNormalized	D_original_ngtdm_Contrast	V_wavelet.HLH_firstorder_Mean
A_wavelet.LHL_firstorder_Skewness	V_wavelet.LHL_glcm_MCC	D_log.sigma.3.0.mm.3D_glszm_LargeAreaLowGrayLevelEmphasis	V_wavelet.HHH_glszm_LargeAreaLowGrayLevelEmphasis
A_wavelet.LHL_gldm_SmallDependenceLowGrayLevelEmphasis	V_wavelet.LHL_glszm_LargeAreaHighGrayLevelEmphasis	D_log.sigma.3.0.mm.3D_gldm_DependenceNonUniformityNormalized	V_wavelet.HHH_gldm_SmallDependenceLowGrayLevelEmphasis
A_wavelet.LHH_glszm_SmallAreaEmphasis	V_wavelet.LHL_gldm_DependenceVariance	D_log.sigma.3.0.mm.3D_ngtdm_Contrast	D_original_firstorder_Range
	V_wavelet.HLL_glszm_SizeZoneNonUniformity	D_log.sigma.5.0.mm.3D_glszm_LargeAreaHighGrayLevelEmphasis	D_original_ngtdm_Contrast
	V_wavelet.HLH_firstorder_Mean	D_log.sigma.5.0.mm.3D_ngtdm_Busyness	D_log.sigma.3.0.mm.3D_glszm_LargeAreaLowGrayLevelEmphasis
	V_wavelet.HHH_glszm_LargeAreaLowGrayLevelEmphasis	D_wavelet.LLH_glcm_InverseVariance	D_log.sigma.3.0.mm.3D_gldm_DependenceNonUniformityNormalized
	V_wavelet.HHH_gldm_SmallDependenceLowGrayLevelEmphasis	D_wavelet.LHL_glszm_GrayLevelNonUniformityNormalized	D_log.sigma.5.0.mm.3D_ngtdm_Busyness
		D_wavelet.LHL_glszm_LargeAreaHighGrayLevelEmphasis	D_wavelet.LLH_glcm_InverseVariance
		D_wavelet.LHH_glcm_InverseVariance	D_wavelet.LHL_glszm_GrayLevelNonUniformityNormalized
		D_wavelet.LHH_glszm_LargeAreaEmphasis	D_wavelet.LHH_glcm_InverseVariance
		D_wavelet.LHH_gldm_DependenceNonUniformityNormalized	D_wavelet.LHH_gldm_DependenceNonUniformityNormalized
		D_wavelet.HLH_glcm_Imc1	D_wavelet.HLH_glcm_Imc1
		D_wavelet.LLL_firstorder_Skewness	D_wavelet.LLL_firstorder_Skewness

**Table 3:** Predictive performance of different models in training and validation cohorts.

Feature_num	Methods	Training cohort						Validation cohort					
		AUC	Accuracy	Sensitivity	Specificity	PPV	NPV	AUC	Accuracy	Sensitivity	Specificity	PPV	NPV
6	APR	0.775(0.715-0.835)	0.698(0.653-0.741)	0.716(0.612-0.806)	0.695(0.583-0.810)	0.296(0.265-0.322)	0.932(0.920-0.941)	0.786(0.644-0.929)	0.689(0.557-0.801)	0.667(0.333-1.000)	0.692(0.519-0.962)	0.273(0.158-0.360)	0.923(0.900-0.943)
10	VPR	0.827(0.774-0.880)	0.744(0.700-0.784)	0.731(0.612-0.836)	0.746(0.631-0.869)	0.340(0.301-0.371)	0.939(0.929-0.948)	0.810(0.674-0.946)	0.754(0.627-0.855)	0.556(0.222-0.889)	0.788(0.500-1.000)	0.312(0.154-0.421)	0.911(0.867-0.929)
16	DPR	0.887(0.847-0.927)	0.787(0.746-0.824)	0.791(0.701-0.896)	0.786(0.722-0.909)	0.398(0.370-0.429)	0.955(0.951-0.960)	0.953(0.903-1.000)	0.852(0.738-0.930)	1.000(0.778-1.000)	0.827(0.808-0.981)	0.500(0.437-0.500)	1.000(1.000-1.000)
16	FR	0.904(0.870-0.938)	0.803(0.762-0.839)	0.836(0.716-0.925)	0.797(0.684-0.885)	0.424(0.387-0.449)	0.964(0.959-0.968)	0.893(0.804-0.982)	0.787(0.663-0.881)	0.778(0.444-1.000)	0.788(0.635-0.962)	0.389(0.267-0.450)	0.953(0.943-0.962)
3	Clinical	0.781(0.722-0.840)	0.721(0.677-0.762)	0.716(0.567-0.836)	0.722(0.618-0.799)	0.316(0.268-0.350)	0.934(0.924-0.940)	0.919(0.833-1.000)	0.869(0.758-0.942)	0.889(0.442-1.000)	0.865(0.596-1.000)	0.533(0.362-0.563)	0.978(0.969-0.981)
4	Clinical Radiomics	0.898(0.860-0.937)	0.837(0.799-0.870)	0.821(0.672-0.896)	0.840(0.663-0.912)	0.478(0.429-0.500)	0.963(0.954-0.966)	0.964(0.919-1.000)	0.918(0.819-0.973)	1.000(0.667-1.000)	0.904(0.846-1.000)	0.643(0.545-0.643)	1.000(1.000-1.000)

FR, fusion of radiomics features of arterial phase, venous phase, and delayed phase; Clinical, fusion of clinical, and radiological characteristics; Clinical Radiomics, fusion of clinicalradiological features and radiomics features. APR, radiomics model of arterial phase; AUC, area under the curve; D, DPR, radiomics model of delayed phase; NPV, negative predictive value; PPV, positive predictive value; VPR, radiomics model of venous phase.

### Predictive Performance of the Combined Model

**Figure 4:**
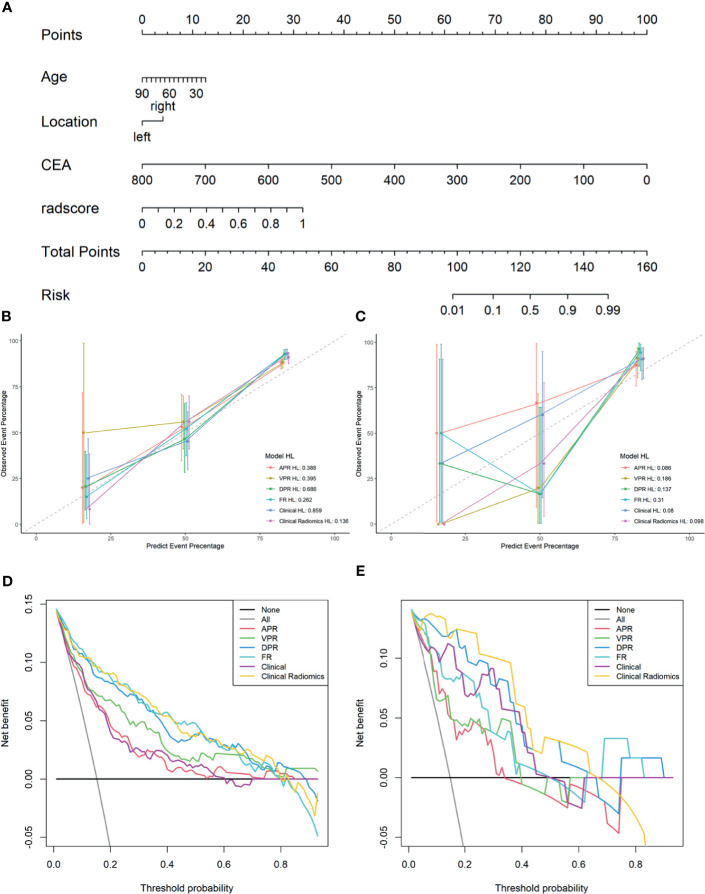
A Clinical Radiomics nomogram for preoperative identification of microsatellite instability status in colorectal cancer patients **(A)**. The nomogram was constructed based on multivariate logistic regression and consisted of three clinical factors and 16 radiomics signatures. Calibration curves of the different models in training cohort **(B)** and validation cohort **(C)**; the y-axis represents the actual microsatellite instability rate and the x-axis represents the predicted microsatellite instability risk. The diagonal dotted line indicates that the predicted outcome perfectly corresponds with the actual outcome. The solid line indicates the bias-corrected accuracy of the different models, with a closer fit to the diagonal dotted line representing a better prediction. Decision curve analysis of the different models in training cohort **(D)** and validation cohort **(E)**; the y-axis represents the net benefit, which is calculated by subtracting the expected harm (false positives) from the expected benefit (gaining true positives) and subtracting expected harm (deleting false positives). The higher curve at any given threshold probability is the optimal prediction to maximize net benefit. The solid colored lines represent the different models. The solid gray line represents the assumption that all patients had microsatellite instability. The solid black line represents the assumption that no patients had microsatellite instability. APR, radiomics model of arterial phase; DPR, radiomics model of delayed phase; VPR, radiomics model of venous phase; FR, radiomics model of fusion of arterial phase, delayed phase and venous phase features; Clinical Radiomics, fusion of clinical risk factors and radiomics features of delayed phase; HL, Hosmer-Lemeshow test.

As shown in **Figure 4A**, a Clinical Radiomics combined model was developed that incorporates clinical risk factors and DP radiomics signatures, which was presented as a quantitative nomogram. The Clinical Radiomics model showed excellent predictive ability for MSI status, with an AUC, accuracy, and sensitivity of 0.898, 0.837, and 0.821 in the training cohort and 0.964, 0.918, and 1.000 in the validation cohort, respectively. As shown in **Table 3** and **Figure 3**, the Clinical Radiomics model had a better predictive AUC value than either the clinical model or the radiomics models in the training cohort and validation cohort.

**Figure 5:**
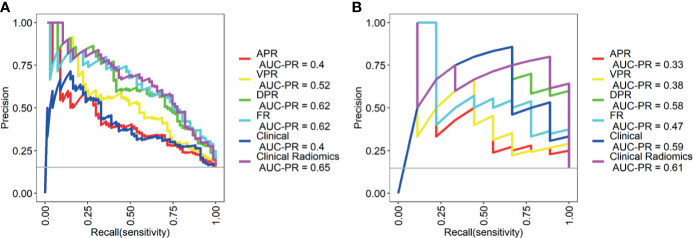
Precision-recall (PR) curves of the different models in the training cohort **(A)** and validation cohort **(B)**. PR represents the relationship between precision and recall. The larger the area under the curve value of the PR curve, the better the model performance. Precision = true positive/(true positive + false positive); recall = true positive/(true positive + false negative). APR, radiomics model of arterial phase; DPR, radiomics model of delayed phase; VPR, radiomics model of venous phase; FR, radiomics model of fusion of arterial phase, delayed phase and venous phase features; Clinical Radiomics, fusion of clinical risk factors and radiomics features of delayed phase.

**Figure 6:**
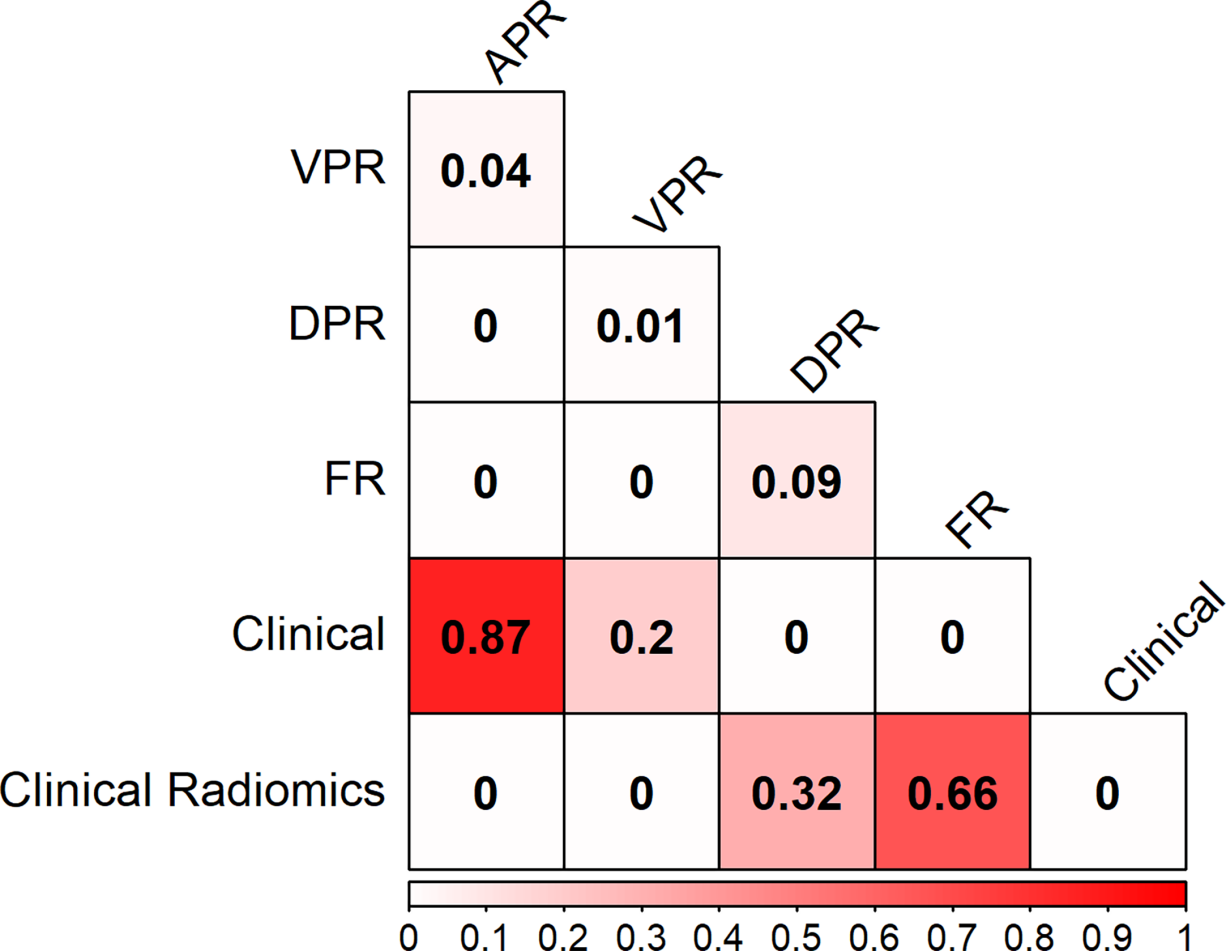
Heat map comparison of the different models in the training cohort. The values in the matrix represent the results of Delong test between two models. APR, radiomics model of arterial phase; DPR, radiomics model of delayed phase; VPR, radiomics model of venous phase; FR, radiomics model of fusion of arterial phase, delayed phase and venous phase features; Clinical Radiomics, fusion of clinical risk factors and radiomics features of delayed phase.

The PR curves show that the Clinical Radiomics model had better MSI prediction performance than the clinical or radiomics models (**Figure 5**). DeLong test results showed a significant difference between the AUC of the Clinical Radiomics model and of the APR, VPR, and clinical models in the training cohort (**Figure 6**). The calibration curve of the nomogram showed favorable agreement between prediction and observation in predicting the risk of MSI (**Figures 4B, C**). The HL test yielded non-significant statistics in the training and validation cohorts, indicating goodness-of-fit in the models.

The DCA results for the clinical model, radiomics models, and combined nomogram are presented in **Figures 4D, E**. The nomogram achieved more clinical utility in predicting MSI than the clinical model or radiomics model alone. The DCA curve of the nomogram demonstrated that when the threshold probability of a patient or doctor ranged between 5% and 80%, the use of the nomogram added greater benefit for MSI prediction than the treat-all-patients scheme or the treat-none scheme in the training cohort.

## Discussion

In this study, we investigated the association between triphasic enhanced CT radiomics features and MSI status. Six, ten, and sixteen radiomics features showed significant correlation with MSI status in AP, DP, and VP, respectively. Four radiomics models (APR, DPR, VPR, and FR) were proposed using the above radiomics features in the training cohort to predict MSI status for patients with colorectal cancer, and we validated its performance in an external validation cohort from another center. Our study showed that the DPR model had a higher outstanding performance than the APR or VPR models in both the training and external validation cohorts. Meanwhile, the nomogram, based on DP radiomics features and clinical risk factors, showed excellent identification ability for MSI status in both training (AUC: 0.898, 95% CI 0.860-0.937) and external validation (AUC: 0.964, 95% CI 0.919–1.000) cohorts. Our nomogram may be useful for predicting the MSI status of CRC patients and, thus, has the potential to aid in the determination of therapeutic strategies. In common studies, the results of external validation cohort are lower than the training cohort due to overfitting. Our results show that the results of external validation cohort are slightly higher than the training cohort, and lack of overlap between the 95% CI of the accuracies between the training and the validation cohorts. Since our validation cohort is external data set, there is often some deviation in distribution between the two data sets due to geographical location and other factors, which may cause the model performance of the validation cohort higher than the training cohort. Validation on additional cohort is required to ensure the model’s reproducible and generalizable.

In the present study, the incidence of MSI was 15.19% (67/441) in the training cohort and 14.75% (9/61) in the external validation cohort, which is consistent with previous literature (32, 33). CRC patients with MSI have distinct prognoses and treatment strategies compared to patients with MSS tumors, including better prognosis and benefits from fluorouracil chemotherapy; moreover, MSI may be a negative marker for immunotherapy. Previous studies (28, 29) have investigated the association between MSI and radiomics features. Fan et al. (28) used CT-based radiomics to predict the MSI status in 119 stage II CRC patients. The predictive AUC of the radiomics model (combination of clinical factors and radiomics features) was 0.752. Pernicka et al. (29) proposed a CT-based radiomics model for the prediction of MSI in stage II–III colon cancer. The combined model (combination of clinical factors and radiomics features) had moderate diagnostic efficacy, with AUC values of 0.80 and 0.79 in the training and validation sets, respectively. Both studies contained small samples and lacked effective validation of external data. Our proposed clinicoradiomics combined model performed better than previous models in both training and external validation cohorts. Therefore, it may be a potential quantitative tool for individualized MSI prediction.

Due to the low incidence of MSI, the data distribution in this study was significantly unbalanced. The unbalanced distribution of data is a common problem in classification. Therefore, the bias adjustment method was used to overcome the training fit error in our study. A previous study used synthetic minority over-sampling technique (SMOTE) methods (28) to resolve data imbalance. The SMOTE method is based on increasing the “artificial” sample to resolve the imbalance of the data set. However, this strategy is prone to model overfitting and is difficult to demonstrate validity.

In our study, 1037 quantitative features were extracted from CT images to build radiomics signatures. During the image preprocessing stage, LoG and wavelet filters (27) were applied to process the original image. Of the 1037 radiomics features, 6, 10, and 16 features were retained in AP, DP, and VP images, respectively, all of which demonstrated high correlations with MSI and were stable across multiple centers. To our surprise, the majority of radiomics features were LoG and wavelet filter features (26/32 in radiomics features) in the present study, which means that LoG and wavelet filters can improve the efficiency of capturing more phenotypic features related to MSI of CRC.

In the present study, the texture feature was the most frequent radiomics feature in triphasic enhanced CT signatures (4/6 in AP, 9/10 in VP, 13/16 in DP). Texture features are microscopic features in an image that have been shown to be highly correlated with tumor heterogeneity (34, 35). However, these features are not easily identified by the human eye and cannot be interpreted as having a clear meaning (36). Our results showed that most texture features were associated with MSI status. Compared with the MSS group, the values of these features were significantly higher in the MSI group, which indicated more homogeneity in the ROI. Our finding is in line with those of previous reports (28, 29) that texture features were also the most frequent radiomics features for MSI prediction. We observed that first-order statistic features including A_wavelet.LHL_firstorder_90Percentile, A_wavelet.LHL_firstorder_Skewness, V_wavelet.HLH_firstorder_Mean, D_original_firstorder_Range, and D_wavelet.LLL_firstorder_Skewness were significantly associated with MSI status, which was consistent with the results of the studies by Fan et al. and Pernicka et al. (28, 29) The results of their studies show that the MSI status is associated with kurtosis and intensity histograms.

Among the triphasic enhanced CT models for the prediction of the MSI status in the training cohort, the DPR model showed the highest performance, with an AUC value of 0.887, followed by 0.827 in the VPR model and 0.775 in the APR model. A similar trend was found in the validation cohort; the predictive AUCs of the DPR, VPR, and APR models were 0.953, 0.810, and 0.876, respectively. Although the VP is the most commonly used phase in gastrointestinal radiomics research, and previous radiomics features for MSI prediction were extracted from portal VP CT images. However, to date, this is the first study to develop a radiomics based model to predict the risk of MSI status in CRC patients based on triphasic enhanced CT with big data. To our surprise, the DPR model showed the best predictive performance in the training and validation cohorts. The triphasic enhanced phase images reflect the uptake and clearance of iodine over time in AP, VP, and DP (37). In AP, the contrast agent is mainly in the intervascular space, which results in focal mucosa enhancement. During VP and DP, the contrast agent is evenly distributed between the intervascular space and the extravascular space, leading to a well-proportioned enhancement (38). The degree of tumor enhancement in AP is positively correlated with the density of microvessels in the tumor, while in VP and DP, the degree of tumor enhancement is related to the content of contrast agent in the tumor interstitial space and vascular space. In addition, CRCs lack normal lymphatic drainage, and the contrast agent tends to remain in the tumor interstitial space for a longer time (38, 39). Therefore, CRCs are significantly enhanced in AP, while VP and DP show continuous enhancement. Previous literature shows that the increase in structure in the enhanced image is proportional to iodine concentration (38). The high content and uniform distribution of contrast agents in tumors may be one of the reasons for the high diagnostic efficiency of the DPR model. This is exactly the same as the number of key features in our study. The numbers of radiomics signatures in DP, VP, and AP were 16, 10, and 6, respectively.

The dynamic changes of CRC from AP, VP to DP showed obvious transmural enhancement from inside the tumor to the outside. For triphasic enhanced CT, AP is mainly used for tumor detection and assessment of the tumor extent along the colorectal wall, VP is used for differentiating CRC from adjacent organs and evaluating lymph nodes, and DP is used to determine the depth of tumor invasion (40). Therefore, the range of tumors detected in DP is larger than that in VP or AP. This means that the ROI delineation range of the DP is the largest during the delineation of the tumor in triphasic enhanced CT images, which is consistent with our observations in the process of delineating tumor ROIs. A positive correlation between increased tumor range and increased diagnostic efficiency has been confirmed by previous studies (41). From the above description, another reason for the high predictive performance of the DPR model could be the large ROI range of tumors in DP images.

Age, location, and CEA were independent predictors of MSI status in the multivariate analysis. CRC patients with an MSI status have distinct clinical characteristics compared to those with MSS tumors, such as a predominance of right-sided colonic tumors, and early age. Our finding is consistent with the results of a previous study (28, 29, 42). CEA levels were significantly lower in the MSI group than in the MSS group, while CA125 was significantly higher in the MSI group than in the MSS group in the present study. A significant correlation between MSI status and the above clinical predictors suggests that genetic alterations may have independent influences on CRC development, thus resulting in distinct tumor biological behavior compared with that of MSS tumors. These parameters could be easily obtained and thus considered as novel approaches for predicting MSI status. Further studies are essential to validate our findings.

As for radiation dose, the average dose length product of triphasic enhanced scans was 1934.76 ± 147.18 mGy*cm, which is slightly higher than the diagnostic reference level for adults (1490 mGy*cm) published by China’s National Health Industry standard (WS/T 637-2018) (43). Application of new techniques such as multi-model iterative reconstruction technology could effectively reduce the radiation dose in clinical practice (44).

Several limitations of our study should be noted. First, 501 patients were excluded because they did not meet the inclusion or exclusion criteria, which inevitably produced selection bias. Second, due to the irregular shape of some tumors, manual segmentation is time-consuming and may have observer variability. In future studies, automated segmentation may be a potential tool to resolve this problem. Third, in this study, we used different imaging instruments and acquisition parameters to complete CT scanning. The influence of different instruments and different parameters on radiomics features is obvious. Therefore, it is important to standardize scanning protocols in different instruments and different institutions.

## Conclusion

In conclusion, we proposed and validated a CT-based radiomics model, incorporating clinical risk factors and radiomics parameters, which showed a relatively high diagnostic performance for the risk prediction of MSI in patients with CRC. This model may be a potential tool for preoperatively identifying the MSI status and can be used in individualized therapeutic strategy planning and prognostic prediction.

## Data Availability Statement

Data are available from the corresponding author upon reasonable request.

## Ethics Statement

The studies involving human participants were reviewed and approved by Lanzhou University Second Hospital medical ethics committee. The ethics committee waived the requirement of written informed consent for participation.

## Author Contributions

Conception and design: JZho, HB, and YC. Collection and assembly of the data: YC and YY. Development of the methodology: JR. Data analysis and interpretation: All authors. Manuscript writing: All authors. All authors contributed to the article and approved the submitted version.

## Funding

This study received funding from the National Natural Science Foundation of China (82071872), Open Fun project of Key Laboratory of Medical Imaging of Gansu Province (GSYX202009), Science and Technology Project of Qinghai Province (No. 2017-SF-158) and Qinghai Provincial Key Clinical Specialty Construction Project.

## Conflict of Interest

Author JR was employed by company GE Healthcare.

The remaining authors declare that the research was conducted in the absence of any commercial or financial relationships that could be construed as a potential conflict of interest.

## Abbreviations

CT, computed tomography; CRC, colorectal cancer; MSI/MSS, microsatellite instability/stability; AP, arterial phase; APR, radiomics model of arterial phase; VP, venous phase; VPR, radiomics model of venous phase; DP, delayed phase; DPR, radiomics model of delayed phase; FR, radiomics model of fusion of arterial phase, delayed phase and venous phase features; ROI, region of interest; LoG, Laplacian of Gaussian; CEA, carcinoembryonic antigen; ROC, receiver operating curve; AUC, area under the curve; DCA, decision curve analysis; PR, precision-recall; CI, confidence interval; PPV/NPV, positive/negative predictive value; HL, Hosmer-Lemeshow.

## References

[1] Gerlinger M Rowan AJ Horswell S Larkin J Endesfelder D Gronroos E. Intratumor Heterogeneity and Branched Evolution Revealed by Multiregion Sequencing. N Engl J Med (2012) 366(10):883–92. 10.1056/NEJMoa1113205 PMC4878653 22397650

[2] Smith G Carey FA Beattie J Wilkie MJV Lightfoot TJ Coxhead J. Mutations in APC, Kirsten-Ras, and P53–Alternative Genetic Pathways to Colorectal Cancer. Proc Natl Acad Sci U S A (2002) 99(14):9433–8. 10.1073/pnas.122612899 PMC123158 12093899

[3] Vogelstein B Fearon ER Hamilton SR Kern SE Preisinger AC Leppert M. Genetic Alterations During Colorectal-Tumor Development. N Engl J Med (1988) 319(9):525–32. 10.1056/NEJM198809013190901 2841597

[4] Battaglin F Naseem M Lenz HJ Salem ME. Microsatellite Instability in Colorectal Cancer: Overview of its Clinical Significance and Novel Perspectives. Clin Adv Hematol Oncol (2018) 16(11):735–45. PMC7493692 30543589

[5] Mcdermott U Longley DB Johnston PG. Molecular and Biochemical Markers in Colorectal Cancer. Ann Oncol (2002) 13(Suppl 4):235–45. 10.1093/annonc/mdf665 12401696

[6] Gelsomino F Barbolini M Spallanzani A Pugliese G Cascinu S. The Evolving Role of Microsatellite Instability in Colorectal Cancer: A Review. Cancer Treat Rev (2016) 51:19–26. 10.1016/j.ctrv.2016.10.005 27838401

[7] Yang G Zheng RY Jin ZS. Correlations Between Microsatellite Instability and the Biological Behaviour of Tumours. J Cancer Res Clin Oncol (2019) 145(12):2891–9. 10.1007/s00432-019-03053-4 PMC6861542 31617076

[8] Sepulveda AR Hamilton SR Allegra CJ Grody W Cushman-Vokoun AM Funkhouser WK. Molecular Biomarkers for the Evaluation of Colorectal Cancer Guideline From the American Society for Clinical Pathology, College of American Pathologists, Association for Molecular Pathology, and American Society of Clinical Oncology. J Clin Oncol (2017) 35(13):1453–86. 10.1200/JCO.2016.71.9807 28165299

[9] Ribic CM Sargent DJ Moore MJ Thibodeau SN French AJ Goldberg RM. Tumor Microsatellite-Instability Status as a Predictor of Benefit From Fluorouracil-Based Adjuvant Chemotherapy for Colon Cancer. N Engl J Med (2003) 349(3):247–57. 10.1056/NEJMoa022289 PMC3584639 12867608

[10] Sargent DJ Marsoni S Monges G Thibodeau SN Labianca R Hamilton SR. Defective Mismatch Repair as a Predictive Marker for Lack of Efficacy of Fluorouracil-Based Adjuvant Therapy in Colon Cancer. J Clin Oncol (2010) 28(20):3219–26. 10.1200/JCO.2009.27.1825 PMC2903323 20498393

[11] Kather JN Pearson AT Halama N Jäger D Krause J Loosen SH. Deep Learning can Predict Microsatellite Instability Directly From Histology in Gastrointestinal Cancer. Nat Med (2019) 25(7):1054–6. 10.1038/s41591-019-0462-y PMC7423299 31160815

[12] Malesci A Laghi L Bianchi P Delconte G Randolph A Torri V. Reduced Likelihood of Metastases in Patients With Microsatellite-Unstable Colorectal Cancer. Clin Cancer Res (2007) 13(13):3831–9. 10.1158/1078-0432.CCR-07-0366 17606714

[13] Sargent DJ Shi Q Yothers G Tejpar S Bertagnolli MM Thibodeau SN. Prognostic Impact of Deficient Mismatch Repair (Dmmr) in 7,803 Stage II/III Colon Cancer (CC) Patients (Pts): A Pooled Individual Pt Data Analysis of 17 Adjuvant Trials in the ACCENT Database. Asco Meeting Abstracts (2014) 32. 10.1200/jco.2014.32.15_suppl.3507

[14] Dienstmann R Mason MJ Sinicrope FA Phipps AI Tejpar S Nesbakken A. Prediction of Overall Survival in Stage II and III Colon Cancer Beyond TNM System: A Retrospective, Pooled Biomarker Study. Ann Oncol (2017) 28(5):1023–31. 10.1093/annonc/mdx052 PMC5406760 28453697

[15] Cutsem V E Cervantes A Adam R. ESMO Consensus Guidelines for the Management of Patients With Metastatic Colorectal Cancer. Ann Oncol (2016) 27(8):1386–422. 10.1093/annonc/mdw235 27380959

[16] Watanabe T Muro K Ajioka Y Hashiguchi Y Ito Y Saito Y. Japanese Society for Cancer of the Colon and Rectum (JSCCR) Guidelines 2016 for the Treatment of Colorectal Cancer. Int J Clin Oncol (2018) 23(1):1–34. 10.1007/s10147-017-1101-6 28349281 PMC5809573

[17] Umar A Boland CR Terdiman JP Syngal S Srivastava S. Revised Bethesda Guidelines for Hereditary Nonpolyposis Colorectal Cancer (Lynch Syndrome) and Microsatellite Instability. J Natl Cancer Inst (2004) 96(4):261–8. 10.1093/jnci/djh034 PMC2933058 14970275

[18] Itakura H Achrol AS Mitchell LA Loya JJ Liu T Westbroek EM. Magnetic Resonance Image Features Identify Glioblastoma Phenotypic Subtypes With Distinct Molecular Pathway Activities. Sci Transl Med (2015) 7(303):303ra138–303ra138. 10.1126/scitranslmed.aaa7582 PMC4666025 26333934

[19] Sacher AG Dahlberg SE Heng J Mach S Jn PA Oxnard GR. Association Between Younger Age and Targetable Genomic Alterations and Prognosis in non–Small-Cell Lung Cancer. JAMA Oncol (2016) 2(3):313–20. 10.1001/jamaoncol.2015.4482 PMC4819418 26720421

[20] Wen-Yue Y Jing H Li X Lei C Mi Y Li L. Prediction of Biological Behavior and Prognosis of Colorectal Cancer Patients by Tumor MSI/MMR in the Chinese Population. Onco Targets Ther (2016) 9:7415–24. 10.2147/OTT.S117089 PMC5153316 27994472

[21] Huang YQ Liang CH He L Tian J Liang CS Chen X. Development and Validation of a Radiomics Nomogram for Preoperative Prediction of Lymph Node Metastasis in Colorectal Cancer. J Clin Oncol (2016) 34(18):2157–64. 10.1200/JCO.2015.65.9128 27138577

[22] Nie K Shi L Chen Q Hu X Jabbour SK Yue N. Rectal Cancer: Assessment of Neoadjuvant Chemoradiation Outcome Based on Radiomics of Multiparametric MRI. Clin Cancer Res (2016) 22(21):5256–64. 10.1158/1078-0432.CCR-15-2997 PMC10916000 27185368

[23] Gillies RJ Kinahan PE Hricak H. Radiomics: Images are More Than Pictures, They are Data. Radiology (2016) 278(2):563–77. 10.1148/radiol.2015151169 PMC4734157 26579733

[24] Zhang S Zhang B Tian J Dong D Gu DS Dong YH. Radiomics Features of Multiparametric MRI as Novel Prognostic Factors in Advanced Nasopharyngeal Carcinoma. Clin Cancer Res (2017) 23(15):4259–69. 10.1158/1078-0432.CCR-16-2910 28280088

[25] Huang X Cheng Z Huang Y Liang C He L Ma Z. CT-Based Radiomics Signature to Discriminate High-Grade From Low-Grade Colorectal Adenocarcinoma. Acad Radiol (2018) 25(10):1285–97. 10.1016/j.acra.2018.01.020 29503175

[26] Horvat N Veeraraghavan H Khan M Blazic I Zheng J Capanu M. MR Imaging of Rectal Cancer: Radiomics Analysis to Assess Treatment Response After Neoadjuvant Therapy. Radiology (2018) 287(3):833–43. 10.1148/radiol.2018172300 PMC5978457 29514017

[27] Cui Y Liu H Ren J Du X Wang D. Development and Validation of a MRI-Based Radiomics Signature for Prediction of KRAS Mutation in Rectal Cancer. Eur Radiol (2020) 30(4):1948–58. 10.1007/s00330-019-06572-3 31942672

[28] Fan S Li X Cui X Zheng L Ren X Ma W. Computed Tomography-Based Radiomic Features Could Potentially Predict Microsatellite Instability Status in Stage II Colorectal Cancer: A Preliminary Study. Acad Radiol (2019) 26(12):1633–40. 10.1016/j.acra.2019.02.009 30929999

[29] Golia Pernicka JS Gagniere J Chakraborty J Yamashita R Nardo L Creasy JM. Radiomics-Based Prediction of Microsatellite Instability in Colorectal Cancer at Initial Computed Tomography Evaluation. Abdom Radiol (NY) (2019) 44(11):3755–63. 10.1007/s00261-019-02117-w PMC6824954 31250180

[30] Aerts HJWL Velazquez ER Leijenaar RTH Parmar C Grossmann P Carvalho S. Decoding Tumour Phenotype by Noninvasive Imaging Using a Quantitative Radiomics Approach. Nat Commun (2014) 5:4006. 10.1038/ncomms5644 24892406 PMC4059926

[31] Griethuysen JJMV Fedorov A Parmar C Hosny A Aerts HJWL. Computational Radiomics System to Decode the Radiographic Phenotype. Cancer Res (2017) 77(21):e104–7. 10.1158/0008-5472.CAN-17-0339 PMC5672828 29092951

[32] Kawakami H Zaanan A Sinicrope FA. Microsatellite Instability Testing and its Role in the Management of Colorectal Cancer. Curr Treat Options Oncol (2015) 16(7):30. 10.1007/s11864-015-0348-2 26031544 PMC4594190

[33] Pino MS Chung DC. The Chromosomal Instability Pathway in Colon Cancer. Gastroenterology (2010) 138(6):2059–72. 10.1053/j.gastro.2009.12.065 PMC4243705 20420946

[34] Liu H Zhang C Wang L Luo R Li J Zheng H. MRI Radiomics Analysis for Predicting Preoperative Synchronous Distant Metastasis in Patients With Rectal Cancer. Eur Radiol (2019) 29(8):4418–26. 10.1007/s00330-018-5802-7 30413955

[35] Meng X Xia W Xie P Zhang R Li W Wang M. Preoperative Radiomic Signature Based on Multiparametric Magnetic Resonance Imaging for Noninvasive Evaluation of Biological Characteristics in Rectal Cancer. Eur Radiol (2019) 29(6):3200–9. 10.1007/s00330-018-5763-x 30413959

[36] Zhang J Yao K Liu P Liu Z Zhou J. A Radiomics Model for Preoperative Prediction of Brain Invasion in Meningioma non-Invasively Based on MRI: A Multicentre Study. EBioMedicine (2020) 58:102933. 10.1016/j.ebiom.2020.102933 32739863 PMC7393568

[37] Wu J Lv Y Wang N Zhao Y Zhang P Liu Y. The Value of Single-Source Dual-Energy CT Imaging for Discriminating Microsatellite Instability From Microsatellite Stability Human Colorectal Cancer. Eur Radiol (2019) 29(7):3782–90. 10.1007/s00330-019-06144-5 30903331

[38] Miles KA. Tumour Angiogenesis and its Relation to Contrast Enhancement on Computed Tomography: A Review. Eur J Radiol (1999) 30(3):198–205. 10.1016/S0720-048X(99)00012-1 10452718

[39] Zhang M Kono M. Solitary Pulmonary Nodules: Evaluation of Blood Flow Patterns With Dynamic CT. Radiology (1997) 205(2):471–8. 10.1148/radiology.205.2.9356631 9356631

[40] Lee JH Jeong YK Kim DH Go BK Woo YJ Ham SY. Two-Phase Helical CT for Detection of Early Gastric Carcinoma: Importance of the Mucosal Phase for Analysis of the Abnormal Mucosal Layer. J Comput Assist Tomogr (2000) 24(5):777–82. 10.1097/00004728-200009000-00020 11045702

[41] Beig N Khorrami M Alilou M Prasanna P Madabhushi A. Perinodular and Intranodular Radiomic Features on Lung CT Images Distinguish Adenocarcinomas From Granulomas. Radiology (2018) 290(3):180910. 10.1148/radiol.2018180910 PMC6394783 30561278

[42] Wu J Zhang Q Zhao Y Liu Y Chen A Li X. Radiomics Analysis of Iodine-Based Material Decomposition Images With Dual-Energy Computed Tomography Imaging for Preoperatively Predicting Microsatellite Instability Status in Colorectal Cancer. Front Oncol (2019) 9:1250. 10.3389/fonc.2019.01250 31824843 PMC6883423

[43] Cao Y Zhang G Bao H Zhang S Zhang J Zhao Z. Development of a Dual-Energy Spectral CT Based Nomogram for the Preoperative Discrimination of Mutated and Wild-Type KRAS in Patients With Colorectal Cancer. Clin Imaging (2020) 69:205–12. 10.1016/j.clinimag.2020.08.023 32920468

[44] Jia Y Zhai B He T Yu Y Yu N Duan H. The Application of a New Model-Based Iterative Reconstruction in Low-Dose Upper Abdominal CT. Acad Radiol (2019) 26(10):e275–83. 10.1016/j.acra.2018.11.020 30660470

