# Supplementary material for: Predicting Microsatellite Instability Status in Colorectal Cancer Based on Triphasic Enhanced Computed Tomography Radiomics Signatures: A Multicenter Study
Source: Front Oncol. 2021 Jun 10;11:687771. doi: 10.3389/fonc.2021.687771 (PMC8222982; doi:10.3389/fonc.2021.687771)
Supplement: Supplementary file 1 [file DataSheet_1.docx]

**Supplementary Materials**

1. **Supplementary methods**
2. **Supplementary reference**
3. **Supplementary figures**
4. **Supplementary tables**

**I．Supplementary methods**

**The specific definition and description of the features were as follows:**

(1) First-order histogram features. These features describe the central tendency, variability, uniformity, asymmetry, skewness and magnitude of the attenuation values in a given region of interest (ROI), disregarding the spatial relationship of the individual voxels.

(2) Morphologic features. Morphologic features describe the size and shape of a given ROI, without taking into account the attenuation values of its voxels.

(3) Gray Level co-existence Matrix (GLCM) features. A GLCM describes the number of times a voxel of a given attenuation value i is located next to a voxel of j.

(4) Gray-level size zone matrix (GLSZM) features. A GLSZM features describes gray level zones in a ROI, which are defined as the number of connected voxels that share the same gray level intensity.

(5) Gray Level range-matrix (GLRM) features. A GLRM features describes gray level runs, which are defined as the length in number of pixels, of consecutive pixels that have the same gray level value.

(6) Neighbouring Gray Tone Difference Matrix (NGTDM) features. A NGTDM features quantifies the difference between a gray value and the average gray value of its neighbours within distance δ.

(7) Gray Level Dependence Matrix (GLDM) features. A GLDM features quantifies gray level dependencies in an image. A gray level dependency is defined as the number of connected voxels within distance δ that are dependent on the center voxel.

**Discrimination**

Receiver Operating Curve (ROC) was plotted to show the performance of our models in predicting *KRAS* mutation in CRC. Discrimination performance of the models was quantified with the area under the ROC (AUC) value in the training cohort and validated in the independent validation cohort.

**Calibration**

Calibration, which measures the model’s ability to generate predictions that are on average close to the average observed outcome, was plotted to explore the predictive accuracy in the training cohort and validation cohort.

The Hosmer-Lemeshow (H-L) test is a statistical test for goodness of fit for logistic regression model. It examines how well the percentage of observed *KRAS* mutation matches the percentage of predicted *KRAS* mutation over deciles of predicted risk. Given our fitted model, using the calculated test statistic, the *P*-value can be calculated as the right-handed tail probability of the corresponding chi-squared distribution. The *P*-values of more than 0.05 were considered well-calibrated and small *P*-value indicates poor fit.

**Decision curve analysis (DCA)**

DCA was conducted to determine the clinical usefulness of the prediction models by deriving the net benefits at different threshold probabilities in the validation cohort, which was yield by plotting net benefit against threshold probability [1].

**Ⅱ. Supplementary reference**

1. Pencina MJ, D'Agostino RB, Sr., D'Agostino RB, Jr, Vasan RS. Evaluating the added predictive ability of a new marker: from area under the ROC curve to reclassification and beyond. Stat Med. 2008;27(2):157-72; discussion 207-12.

**Ⅲ. Supplementary Figures**





**Supplementary Fig. 1.** Boxplots (A–P) of the 16 radiomics features with significant differences between the microsatellite instability and microsatellite stable groups in the training dataset. MSI, microsatellite instability; MSS, microsatellite stable.


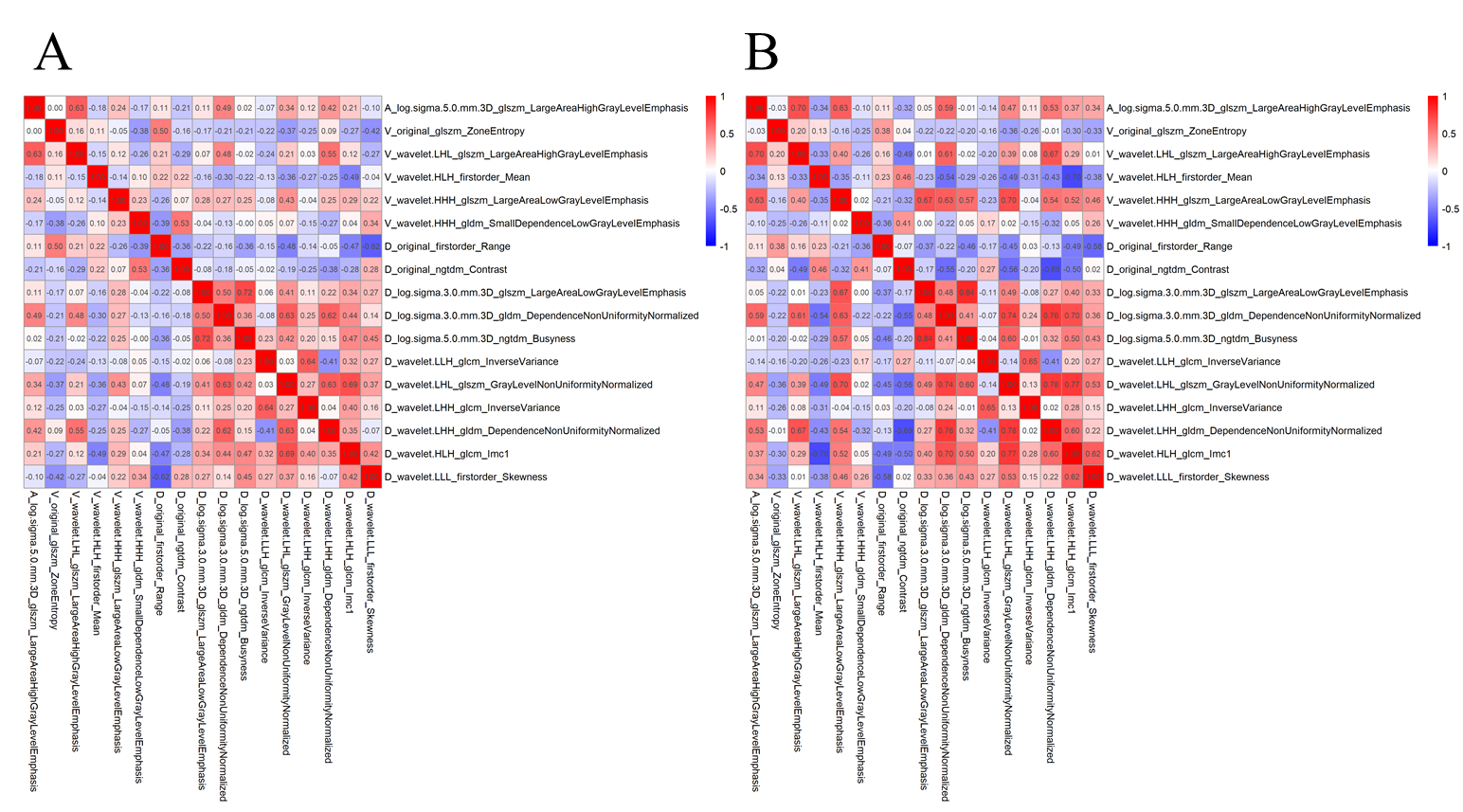


**Supplementary Fig. 2.** Correlation heat map. Seventeen features were highly relevant to microsatellite instability status after combining the radiomics features of the arterial, venous, and delayed phases (FR model). Spearman correlation coefficients represent correlations between features, as shown in the heat map. Dark red indicates a positive correlation, while dark blue indicates a negative correlation. The deeper the color, the stronger the relationship. FR, radiomics model of fusion of arterial phase, delayed phase and venous phase features.

**Ⅳ. Supplementary Tables**

**Table S1.** Scanning parameters of abdominal triphasic enhanced CT in different instruments.

| Parameter | Discovery CT 750 HD | Philips ICT 128 | Siemens SOMATOM Definition Flash 64 |
| --- | --- | --- | --- |
| Tube voltage, kVp | 120 | 120 | 100 |
| Tube current, mAs | 350 | 251 | 145 |
| Collimator width, mm | 40 | 80 | 64×0.6 |
| Rotation time, s | 0.5 | 0.5 | 0.5 |
| Screw pitch, mm | 0.984:1 | 1.150:1 | 0.6 |
| Reconstructed layer thickness, mm | 1.25 | 1.25 | 1.00 |

**Table S2.** The acquisition time of triphasic enhanced CT in different instruments.

| Enhanced phase | Discovery CT 750 HD | Philips ICT 128 | Siemens SOMATOM Definition Flash 64 |
| --- | --- | --- | --- |
| Arterial phase, s | 25-30 | 25-30 | 25 |
| Venous phase, s | 60-70 | 60-70 | 55 |
| Delayed phase, s | 120-150 | 120-150 | 125 |

**Table S3.** Results of multivariate analysis for predicting microsatellite instability status.

| Variables | *P* value | OR | 95% CI |
| --- | --- | --- | --- |
| Age | 0.007 | 0.967 | 0.943-0.991 |
| Location (Left/Right) | 0.034 | 2.144 | 1.055-4.351 |
| CEA | 0.122 | 0.977 | 0.944-0.999 |
| Radscore | <0.001 | 382.3 | 84.7-2023.32 |
